# Determinants of Appointment Planning in Physical Therapy: Insights from Saudi Arabia

**DOI:** 10.3390/healthcare13080893

**Published:** 2025-04-13

**Authors:** Saad A. Alhammad, Omar Khalid Almuhanna, Abdulaziz Riyadh Aljumaah, Muteb Safar Aldosari

**Affiliations:** Department of Rehabilitation Health Sciences, College of Applied Medical Sciences, King Saud University, P.O. Box 10219, Riyadh 11433, Saudi Arabia; ptalmuhanna@outlook.com (O.K.A.); aaljumaahpt@gmail.com (A.R.A.); ptmaldosari@outlook.sa (M.S.A.)

**Keywords:** physical therapy, schedules and appointments, Saudi Arabia, outpatient clinics, hospital, patient compliance, professional autonomy, insurance, rehabilitation

## Abstract

**Background/Objectives:** Appointment planning in physical therapy (PT) is crucial for optimizing patient outcomes and resource efficiency, yet determinants of these plans and deviations from them remain underexplored. This study aimed to explore how physical therapists in Saudi Arabia determine appointment numbers, their preferred planning methods, and the prevalence and contributing factors of deviations from planned appointments. **Methods:** A cross-sectional observational study was conducted using an electronic questionnaire distributed to PTs practicing in outpatient departments and homecare settings across Saudi Arabia. Descriptive statistics were used to summarize therapists’ methods, preferences, and the prevalence of and potential reasons for deviations. **Results:** A total of 434 responses were collected. Most therapists (66%) relied on their evaluation to determine the number of appointments, and this was their preferred method (76%). However, 50% reported patients usually requiring more appointments than initially planned, and 14% did not complete all the planned appointments. Faster-than-expected progress (61%) and slower-than-expected progress (58%) were the primary reasons for deviations. **Conclusions:** Despite most therapists determining the number of appointments based on their evaluation, the majority reported usual deviations from planned appointments, highlighting a gap in appointment planning. Future research should investigate the impact of deviations on patient outcomes and healthcare costs. Strategies to reduce deviations, such as improving adherence to clinical practice guidelines (CPGs), are warranted.

## 1. Introduction

Physical therapy (PT) is a healthcare profession dedicated to enhancing and restoring movement and function, improving quality of life, and preventing disability through evidence-based care [1,2,3,4]. According to the World Confederation for Physical Therapy, physical therapists (PTs) are qualified to assess patients, develop diagnoses and prognoses, and design comprehensive care plans based on examination findings [1]. Similarly, the American Physical Therapy Association emphasizes that PTs evaluate patients and develop treatment plans to restore movement and function [2]. A central aspect of physical therapy is the design and management of individualized treatment strategies that align with each patient’s therapeutic goals and prognosis. This often includes considerations such as the number of appointments required to implement these strategies effectively.

Appointment planning plays a critical role in ensuring these strategies are delivered effectively, as it directly impacts patient outcomes, operational efficiency, and resource utilization [5,6,7,8]. Optimized scheduling has been shown to enhance care delivery and reduce inefficiencies in outpatient services, as evidenced by studies demonstrating the impact of scheduling on clinic flow and patient waiting times [9]. Additionally, shorter waiting times and a greater number of treatment sessions have been associated with better clinical outcomes, including reduced pain and improved functional recovery [10]. However, administrative constraints, such as workplace-imposed session limits, insurance coverage policies, and organizational guidelines, may restrict therapists’ ability to independently determine the number of appointments needed for their patients. These limitations can reduce flexibility and complicate efforts to balance institutional priorities with personalized care.

Deviations from planned appointments, where patients either complete fewer or require more appointments than initially planned, challenge the achievement of treatment goals and efficient resource use. Fewer appointments than planned may result from faster recovery, adherence issues, or external factors such as financial constraints or transportation barriers [7]. Studies in Saudi Arabia have reported similar patterns, with 36% of orthopedic surgery patients and 60% of female patients with lower back pain failing to complete their planned PT appointments, often due to financial or access-related barriers [11,12]. Similarly, other Saudi studies have identified transportation difficulties, ongoing pain, and lack of engagement as common obstacles to adherence, while forgetfulness has been cited as a frequent reason for missed outpatient appointments [13,14]. These deviations can disrupt care continuity, lead to underutilized resources, and compromise treatment outcomes, posing significant challenges in outpatient physiotherapy settings by reducing effectiveness and straining clinic efficiency [15,16,17]. Additional appointments may arise due to slower recovery or unforeseen complications, reflecting variability in patient progress and therapeutic needs. Such deviations highlight the complexity of scheduling in physical therapy, yet little is known about their prevalence or contributing factors. Addressing this gap is essential to optimize appointment planning and care delivery.

Variability in the number of appointments has been documented [18], and while prior research has explored the average number of appointments required for specific conditions [19,20,21], the methods used by physical therapists to determine these numbers remain underexplored, and unlike previous studies that primarily focused on patient adherence and reported barriers to attendance, this study shifts the focus to physical therapists’ perspectives on appointment planning and deviations, providing a more comprehensive understanding of appointment management in Saudi outpatient physical therapy settings.

This study aims to address these gaps by exploring the following:

- How physical therapists determine the number of appointments for their patients and exploring their preferences for appointment planning methods.
- The prevalence of deviations from the planned number of appointments and identifying factors contributing to these deviations, as reported by physical therapists.

## 2. Materials and Methods

This study used an observational, cross-sectional design. Data were collected using an electronic questionnaire distributed to PTs practicing in outpatient departments (OPDs) and homecare services across Saudi Arabia (SA). Participants were included if they were currently practicing PT in OPDs or homecare services in SA. Participants were excluded if they were practicing in settings other than OPDs or homecare services. The questionnaire was disseminated using social media platforms and in-person visits to different hospitals and PT centers. Data were collected from 25 February to 26 May 2024. Descriptive statistics were calculated using Microsoft Excel to summarize the data. The study was conducted in accordance with the Declaration of Helsinki and approved by the Institutional Review Board of King Saud University Institutional Review Board (KSU-IRB#E-24-8553, 6 February 2024).

The questionnaire was pilot-tested on five PT experts from different subspecialties. They reviewed each item to ensure clarity, relevance, and appropriateness. Their feedback was incorporated before the final dissemination. The questionnaire consisted of two sections. The first section included demographic data, such as age, sex, nationality, years of experience, and work setting. The second section focused on appointment planning and included three to four items, where participants were asked about their current and preferred methods for determining the number of appointments for their patients, whether patients usually complete the planned number of appointments or deviate from the plan, and the reasons reported for such deviations. The questionnaire included multiselect questions, allowing participants to choose more than one response, as well as multiple-choice questions.

## 3. Results

### 3.1. Participants’ Details

A total of 514 PTs participated, with 80 excluded for not meeting the inclusion criteria, resulting in a final sample of 434 participants. The participants’ details are summarized in Table 1. The majority were female (56%, n = 241), and the largest age group was 25–29 years (55%, n = 238). Most participants were Saudi (93%, n = 405) and held a bachelor’s degree as their highest level of education (84%, n = 363). The majority had 1–4 years of experience (68%, n = 296), worked in private settings (59%, n = 258), and practiced predominantly in the central region (55%, n = 240). Additionally, 67% (n = 291) reported musculoskeletal conditions as the primary issue of their patient population. Regarding patient volume, nearly half (48%, n = 208) reported seeing 5–8 patients daily.

### 3.2. How Therapists Determine the Number of Appointments

**Figure 1:**
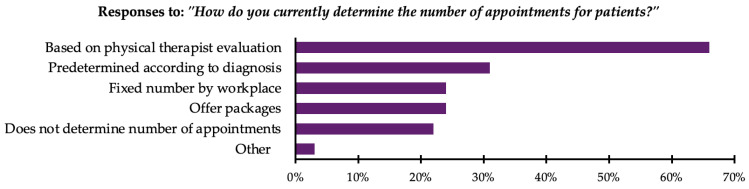
Methods used by therapists to determine number of appointments (n = 434).

In response to the question “How do you currently determine the number of appointments for patients?”, participants were allowed to select multiple options. The majority, 66% (n = 286), reported that the number of appointments was based on the therapist’s evaluation. Moreover, 32% (n = 139) reported that a predetermined number of appointments was used based on the diagnosis. Additionally, 24% (n = 102) reported that their workplace set a fixed number of appointments regardless of evaluation or diagnosis. Another 24% (n = 105) reported offering appointment packages, where a specific number of appointments are provided at a discounted price. Furthermore, 22% (n = 97) reported that they do not predetermine the number of appointments, choosing instead to proceed on an appointment-by-appointment basis. Finally, 3% (n = 15) selected “other”, reporting practices such as providing an “infinite number” of appointments until the patient decides to stop, relying on doctor’s referrals and orders, or determining appointments based on whether the patient is insured or paying in cash. All responses are presented in Figure 1.

### 3.3. Therapists’ Preferred Methods of Planning Appointments

**Figure 2:**
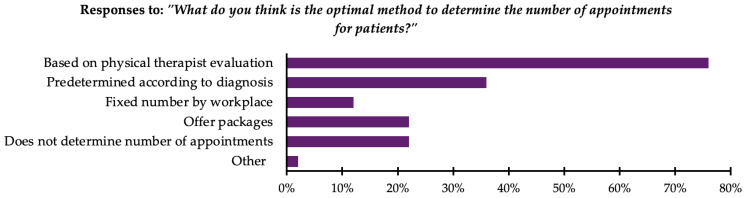
Preferred methods used by therapists to determine number of appointments (n = 434).

In response to the question “What do you think is the optimal method to determine the number of appointments for patients?”, participants were allowed to select multiple options. The majority, 76% (n = 332), reported that the number of appointments should be based on the therapist’s evaluation. Moreover, 36% (n = 155) reported using a predetermined number of appointments based on the diagnosis. Additionally, 22% (n = 95) reported offering appointment packages, where a specific number of appointments are provided at a discounted price. Another 22% (n = 95) reported that the number of appointments should not be predetermined, choosing instead to proceed on an appointment-by-appointment basis. Furthermore, 12% (n = 51) reported that a fixed number of appointments should be set by their workplace regardless of evaluation or diagnosis. Finally, 2% (n = 9) selected “other”, reporting practices such as determining the number of appointments based on protocols or patient load at the clinic. All responses are presented in Figure 2.

### 3.4. Prevalence of Deviations from the Planned Number of Appointments

**Figure 3:**
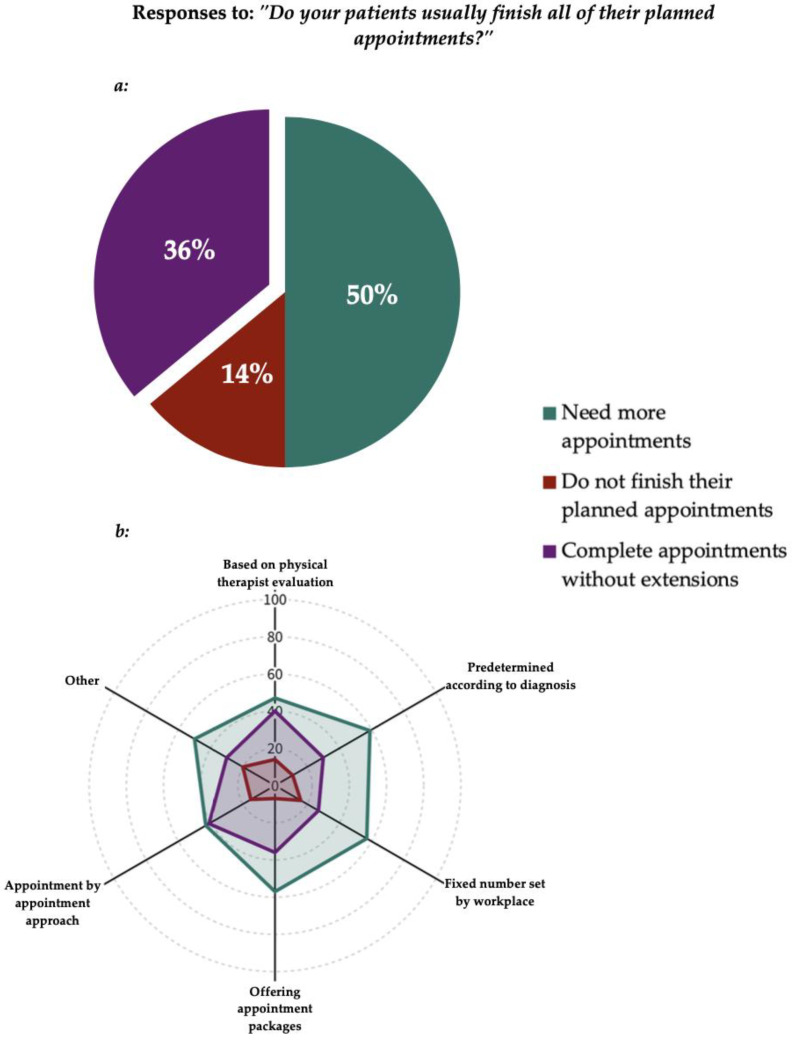
(**a**) Deviations from the planned number of appointments (n = 434). (**b**) Appointment planning methods in relation to deviations, expressed as percentages. The legend applies to both figures.

In response to the question “Do your patients usually finish all of their planned appointments?”, participants were allowed to select one option only. Fifty percent of the respondents, 50% (n = 217), reported that their patients finish all their planned appointments but require additional appointments beyond the planned number. A smaller proportion, 36% (n = 156), reported that their patients finish all their planned appointments without needing any additional appointments. Finally, 14% (n = 61) indicated that their patients usually do not finish all their planned appointments. All responses are presented in Figure 3.

### 3.5. Reasons Reported for Deviations from the Planned Number of Appointments

**Figure 4:**
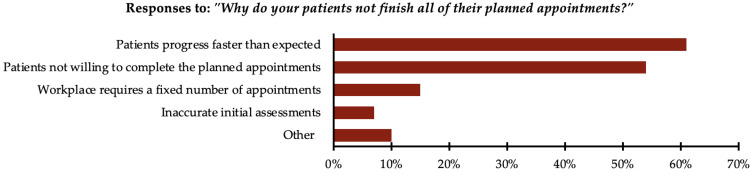
Reasons reported for patients not completing planned number of appointments (n = 61).

Participants who indicated that their patients usually do not finish all their planned appointments were asked a follow-up question: “Why do your patients not finish all of their planned appointments?” Participants were allowed to select multiple options. The majority, 61% (n = 37), reported that patients’ progress was faster than expected. Moreover, 54% (n = 33) reported that patients were not willing to complete the planned appointments. Additionally, 15% (n = 9) reported that their workplace requires a fixed number of appointments. Furthermore, 7% (n = 4) reported inaccurate initial assessments as a contributing factor. Finally, 10% (n = 6) selected “other”, reporting reasons such as financial constraints, accessibility limitations, a lack of understanding about the treatment process and expectations for immediate results, or insurance companies mandating a fixed number of appointments. All responses are presented in Figure 4.

**Figure 5:**
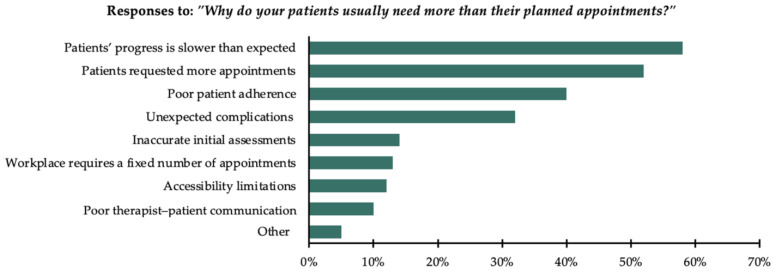
Reasons reported for patients requiring more appointments than the planned number of appointments (n = 217).

Participants who indicated that their patients usually finish all their planned appointments but need additional appointments were asked a follow-up question: “Why do your patients usually need more than their planned appointments?” Participants were allowed to select multiple options. The majority, 58% (n = 126), reported that patients’ progress was slower than expected. Moreover, 52% (n = 112) reported that patients requested more appointments. Additionally, 40% (n = 86) reported poor patient adherence as a contributing factor. Furthermore, 32% (n = 70) reported unexpected complications or setbacks in the patient’s condition. A smaller proportion, 14% (n = 30), reported inaccurate initial assessments; 13% (n = 29), that their workplace requires a fixed number of appointments; 12% (n = 25), accessibility limitations; and 10% (n = 21), poor therapist–patient communication. Finally, 5% (n = 11) selected “other”, reporting reasons such as their workplace requiring an “infinite number” of appointments, the ongoing need for follow-up with lymphoedema patients, slower progress in neurological and pediatric cases, and the presence of insurance coverage. Other reasons included therapists initially setting a lower number of appointments to build patient trust or to evaluate their motivation and response before recommending additional appointments. All responses are presented in Figure 5.

## 4. Discussion

The current study describes how physical therapists determine the number of appointments, their preferences for appointment planning methods, the prevalence of deviations from planned appointments, and the reasons reported for these deviations. Therapist’s evaluation was the most frequently used method for determining the number of appointments, with fewer participants adopting an appointment-by-appointment approach. Similarly, therapist’s evaluation was identified as the preferred method for appointment planning, while a fixed number of appointments set by the workplace was the least preferred. Notably, over half of the participants reported deviations from planned appointments. Half of PTs reported that patients complete their planned appointments but often require additional appointments, while a smaller proportion noted that patients do not finish all their planned appointments. Faster-than-expected progress was the most frequently cited reason for incomplete appointments, whereas slower-than-expected progress was the primary reason for requiring additional sessions. Inaccurate initial assessments and poor therapist–patient communication were among the least cited reasons for these deviations.

Physical therapists in this study reported using their evaluation as the most frequent method for determining the number of appointments and identified this as their preferred approach. The reliance on therapist evaluation emphasizes the importance of PTs’ ability to assess and predict patient outcomes accurately. Cook et al. highlighted that experienced PTs are effective at predicting recovery and disability outcomes, with clinical judgment serving as a significant predictor of recovery rates for conditions such as back and neck pain [22]. Similarly, PTs’ intuition has been affirmed as a good marker of prognosis and patient outcomes [23]. In contrast, a fixed number of appointments set by the workplace was the least preferred method. This finding may highlight the significance of autonomy in PT practice, as it enables therapists to make clinical decisions tailored to individual patient needs. Globally recognized standards in PT emphasize that clinical judgment and independent decision-making are fundamental to effective patient care [1,2,3,4]. Previous research has shown that reduced autonomy is a significant factor contributing to burnout, with PTs experiencing low self-esteem, stress, and even withdrawal from the profession when they lack control over their work [24,25]. Notably, some participants in our sample reported that the number of appointments is determined by the referring physician, further limiting PTs’ control over appointment planning. This restriction directly contradicts the American Physical Therapy Association’s definition of autonomous practice, which is characterized by “independent, self-determined professional judgment and action”, with one of its core objectives being direct patient access to PT services [26]. If such practices still exist, achieving the goal of direct access to PT in Saudi Arabia may remain challenging. Hence, the preference for therapist evaluation over workplace-mandated appointment planning highlights the value of autonomy in PT practice, which not only enhances patient outcomes but also plays a crucial role in therapist job satisfaction and burnout prevention.

Despite the majority of physical therapists relying on their evaluations to plan the number of appointments, most reported deviations from the initial plan, with patients frequently requiring more appointments. This may be influenced by the characteristics of our sample, as 68% of PTs had 1–4 years of experience, a period during which essential skills such as pattern recognition and clinical judgment are still developing, both of which are critical for making accurate prognoses and guiding patient care management [22,23,27,28]. May et al. (2010) reported that pattern recognition was virtually absent in inexperienced physical therapists, and the majority of them lacked a sophisticated clinical reasoning process [29]. A systematic review by Zadro et al. (2019) concluded that many PTs do not follow clinical practice guidelines (CPGs) [30], which likely contributes to slower-than-expected progress, the most reported reason for additional appointments in this study. Supporting this, Rutten et al. (2010), Childs et al. (2015), and Hanney et al. (2016) established that adherence to guideline-based care is associated with fewer treatment sessions, better outcomes, and reduced costs [31,32,33]. In Saudi Arabia, Moslem et al. (2020) found an overall low adherence to CPGs among Saudi PTs [34]. Additionally, Aljohani et al. (2024) noted that knowledge and beliefs of PTs in Saudi Arabia are not aligned with current CPGs in managing patellofemoral pain [35]. Therefore, improving adherence to clinical practice guidelines could help minimize these deviations by aligning treatment approaches with evidence-based care, ultimately reducing the need for additional appointments and enhancing patient outcomes.

While deviations from planned appointments may be influenced by therapists’ adherence to CPGs, patient-related factors also play a role. Low self-efficacy has been identified as a barrier to treatment adherence [36], which aligns with the finding that PTs reported “poor patient adherence” as a reason for patients requiring additional appointments. Patients with low confidence in overcoming obstacles are less likely to follow prescribed rehabilitation plans, potentially increasing their reliance on PTs [37]. This reliance may also contribute to deviations, as PTs cited “patient requested more appointments” as another reason for requiring additional appointments. Conversely, higher levels of self-efficacy have been associated with better perceived clinical improvements and a lower number of required therapy sessions [38]. Thus, enhancing patient self-efficacy may be a key strategy to improving adherence to treatment plans, potentially reducing deviations from planned appointments and minimizing the need for additional therapy appointments.

The existing literature has reported high non-compliance rates in physical therapy attendance. Al-Eisa et al. (2010) found that 60% of Saudi female patients with lower back pain did not complete their scheduled PT sessions [12]. Similarly, Alexandre et al. (2002) reported that 49% of patients with back pain had low compliance with attending their scheduled sessions [39]. Kattan et al. (2023) observed that 36% of patients who underwent orthopedic surgery were non-compliant with PT, including in terms of attendance [11]. Likewise, Friedrich et al. (1998) reported that 51% of chronic lower back pain patients failed to attend all ten prescribed PT sessions [40]. Our study found that only 14% of PTs reported that their patients usually do not complete their planned appointments, which is lower than the percentages reported in previous studies. This difference may be influenced by variations in study populations, treatment settings, and reporting methods. Unlike previous studies that examined specific conditions such as lower back pain or post-surgical rehabilitation, our study encompassed a diverse patient population across multiple musculoskeletal, neurological, and other conditions in outpatient and homecare settings.

Administrative constraints, such as insurance coverage, may also impact appointment planning and contribute to deviations from planned care. Insurance limitations often restrict timely access to physical therapy services, particularly for conditions such as anterior cruciate ligament reconstruction and rotator cuff repair [41,42]. A recent analysis of insurance plans in the United States found that most insurance plans do not provide sufficient coverage for the number of visits needed to achieve full recovery from common orthopedic pathologies [43]. On the other hand, insured patients in Romania were reported to over-utilize healthcare services, often opting for more comprehensive or extended care due to the financial protection offered by insurance [44]. In Saudi Arabia, the healthcare system is predominantly government-funded, providing free healthcare services to citizens through public hospitals and clinics [45]. However, with ongoing healthcare transformation under Vision 2030, insurance-based access to care is expanding, increasing private sector utilization [46]. Uniquely, in this study, a small proportion of participants noted that insurance constraints influenced appointment planning, with decisions often based on whether the patient was insured or paying in cash. Some PTs reported that insurance played a role in appointment deviations. In some cases, reasons for patients requiring additional appointments were simply because “the patient has insurance”, while in others, patients did not complete all planned appointment due to insurance-mandated limits on the number of appointments. These findings highlight how insurance coverage can influence both the overutilization and underutilization of PT services, impacting treatment continuity and resource allocation.

The present study provides valuable insights into an underexplored topic in physical therapy, specifically the determinants of appointment planning and deviations from planned appointments. Another strength of this study is the relatively large sample size compared to studies with the same design. Limitations must be acknowledged. The study may be affected by recall bias from self-reported data, which could impact the accuracy and generalizability of the findings.

## 5. Conclusions

The current study found that therapists primarily rely on and prefer their own evaluation to determine the number of appointments needed by patients. Despite this, the majority (64%) of therapists reported deviations from planned appointments, with 50% indicating that their patients required additional appointments. Future research should investigate the impact of these deviations on patient outcomes, healthcare costs, and their prevalence on specific settings and patient populations. Strategies to minimize the rate of deviations should be developed, which could include enhancing PTs’ adherence to CPGs. Our study provides an overview of an unexplored area in physical therapy, establishing a foundation for future research on appointment planning in Saudi Arabia. A key area for further investigation is understanding why half of patients require additional appointments. Future studies should adopt analytical designs to explore relationships in depth and develop evidence-based strategies to optimize appointment planning.

## Figures and Tables

**Table 1:** Participants’ demographics.

	Number (n)	Percentage (%)
Gender		
Male	193	44
Female	241	56
Age (years)		
20–24	63	15
25–29	238	55
30–34	82	19
35–39	32	7
≥40	19	4
Nationality		
Saudi	405	93
Non-Saudi	29	7
Highest educational level		
Bachelor’s	363	84
Master’s	59	14
PhD	12	3
Years of experience (years)		
1–4	296	68
5–8	78	18
≥9	60	14
Work setting		
Private ^1^	258	59
Public ^2^	133	31
Other ^3^	43	10
Employment region		
Central	240	55
Western	98	23
Eastern	47	11
Southern	35	8
Northern	17	4
Primary patient population seen by the therapist		
Musculoskeletal	291	67
Pediatric	54	12
Sports	45	10
Neurology	30	7
Other ^4^	17	4
Number of patients seen daily		
1–4	55	13
5–8	208	48
9–12	97	22
≥13	74	17

^1^ Public: outpatient services in public hospitals or public clinics. ^2^ Private: outpatient services in private hospitals or private clinics. ^3^ Other (work setting): homecare, daycare centers, and mixed public–private settings. ^4^ Other (primary patient population seen by the therapist): general or mixed practice, cardiopulmonary and vascular, lymphedema, oncology, vestibular, and women’s health.

## Data Availability

The data that support the findings of this study are available on reasonable request from the corresponding author.

## Abbreviations

The following abbreviations are used in this manuscript:

PT	Physical therapy
PTs	Physical therapists
SA	Saudi Arabia
CPGs	Clinical practice guidelines
